# Classifying recurrent *Mycobacterium tuberculosis* cases in Georgia using MIRU-VNTR typing

**DOI:** 10.1371/journal.pone.0223610

**Published:** 2019-10-18

**Authors:** Nino Maghradze, Levan Jugheli, Sonia Borrell, Nestani Tukvadze, Rusudan Aspindzelashvili, Zaza Avaliani, Klaus Reither, Sebastien Gagneux

**Affiliations:** 1 Swiss Tropical and Public Health Institute, Basel, Switzerland; 2 University of Basel, Basel, Switzerland; 3 National Center for Tuberculosis and Lung Diseases (NCTLD), Tbilisi, Georgia; Fundació Institut d’Investigació en Ciències de la Salut Germans Trias i Pujol, Universitat Autònoma de Barcelona, SPAIN

## Abstract

**Introduction:**

Recurrent tuberculosis (TB) is one of the main challenges in TB control. Genotyping based on Mycobacterial Interspersed Repetitive Units–Variable Tandem Repeats (MIRU-VNTR) has been widely used to differentiate between relapse and reinfection, which are the two main causes of recurrent TB. There is a lack of data regarding the causes of TB recurrence in Georgia, and while differentiating between relapse and reinfection plays a key role in defining appropriate interventions, the required genotyping methodologies have not been implemented. The objective of this study was to implement MIRU-VNTR genotyping at the National Center for Tuberculosis and Lung Diseases (NCTBLD) and differentiate between relapse and reinfection in multidrug resistant (MDR-) TB patients from Tbilisi, Georgia.

**Methods:**

Recurrent MDR tuberculosis cases from 2014–2016 diagnosed at NCTLD were included in the study when bacterial samples from both episodes were available. Genotyping based on the MIRU-VNTR 24 loci was implemented and used for differentiating between relapse and reinfection. Paired samples showing the same MIRU-VNTR pattern or one locus difference were classified as relapse, while two and more loci differences were treated as reinfection. Exact logistic regression was used to identify predictors of recurrence.

**Results:**

Thirty two MDR-TB patients (64 samples) were included and MIRU-VNTR 24 typing was performed on the corresponding paired samples. Of the 32 patients, 25 (83.3%) were identified as relapse while 5 (16.7%) were due to re-infection. Patients with a history of incarceration were significantly associated with TB reinfection (p< 0.05).

**Conclusion:**

Recurrent TB in MDR patients in Georgia are mainly caused by relapse, raising concerns on the efficacy of the TB control program. An association between incarceration and reinfection likely reflects high levels of ongoing TB transmission in prisons, indicating the need for better TB infection control measures in these settings. Our results add to the rationale for implementing genotypic surveillance of TB more broadly to support TB control in Georgia.

## Introduction

Tuberculosis (TB) remains a major global health problem [1]. Recurrent TB, defined as a second episode of TB disease in patients previously declared as cured or with successful treatment completion, contributes to the global burden of TB, and thus needs to be properly addressed if TB is to be eliminated [1]. Recurrent TB is caused by two fundamentally different mechanisms, i) relapse caused by the same strains of *Mycobacterium tuberculosis* and ii) exogenous reinfection with a different strain [2].

Relapse refers to the reactivation of a subset of bacteria that have not been successfully eliminated during patient treatment. The underlying causes of relapse are manifold, and include various bacterial and host factors [3]. For example, phenotypic drug tolerance in bacterial persisters and differences in pharmaco-genetic characteristics of patients influence to likelihood of relapse [4,5]. Relapse is thought to be the main contributor to recurrent TB in low incidence areas [6–8]. In contrast, exogenous reinfection with a distinct *M*. *tuberculosis* strain is a particular problem in high incidence countries [9,10]. In addition, several other risk factors such as HIV-infection have been associated with recurrent TB disease [10,11]. Understanding the causes and risk factors driving recurrent TB in a specific epidemiological setting has important implications for defining adequate control strategies [2].

Georgia, located at the border of Eastern Europe and Western Asia, is a TB middle incidence country with an incidence rate of 84/100,000 per year. Multi-drug resistant / Rifampicin-resistant (MDR/RR) TB patients comprise 11% of all new TB cases and 30% of previously treated cases, translating into an incidence of MDR/RR TB of 19/100 000. The rate of recurrent TB cases has been increasing from 10.2 to 15.9 in 2014 and 2017, respectively, mainly due to lost to follow-up patients, especially for MDR and XDR TB [1]. Despite of an overall decline in TB incidence from 228/100,000 in 2002 to 84/100,000 in 2018, Georgia remains one of the high MDR TB burden countries, nowadays showing 56% of successful outcome for 2^nd^ line treatment [1]. From 2013 until 2016, the proportion of lost to follow-up patients enrolled into second-line treatment in Georgia has almost halved, from 32% to 18%, but still remains high. Lost to follow-up patients often suffer from unfavorable outcomes, including a high rate of recurrent TB [8].

Currently, there is no data on the main causes of recurrent TB in Georgia. Moreover, most studies on recurrent TB to date in similar settings have only considered small numbers of drug-resistant patients [6,7,12]. Here we implemented MIRU-VNTR typing in Georgia for the first time and used this technique to differentiate between the two major causes of recurrent disease in MDR TB cases in Georgia. We then tested for potential risk factors associated with either of these two causes of recurrent MDR-TB.

## Materials and methods

### Data source and study design

In Georgia, the National Tuberculosis Program (NTP), established in 1996, covers the whole country and includes 64 TB units, 11 microscopy laboratories, and the National Reference Laboratory (NRL) at the National Center for Tuberculosis and Lung Diseases (NCTBLD) in Tbilisi. The NRL is the only laboratory in the country with capacity to perform phenotypic *M*. *tuberculosis* drug susceptibility testing (DST). All TB cases diagnosed by smear microscopy and/or GeneXpert are sent to the NRL for DST. In addition, the active surveillance of the NTP in Georgia collects standard epidemiological and clinical information for every suspected TB case in the country, including from the civil sector and penitentiary system.

For the purpose of this study, we enrolled a retrospective cohort including bacteriologically confirmed recurrent MDR TB cases recruited between 2014 and 2016. Recurrent TB cases were identified through the National TB Surveillance electronic database and were classified as “successfully treated” (including treatment completion) and “lost to follow-up” depending on the outcome of the primary TB episode. Two cultures per patient and the related patient information were included in the study.

### Ethics

The study was conducted at the National Reference Laboratory (NRL) in collaboration with the Swiss Tropical and Public Health Institute. Ethical approval was obtained by the relevant authorities in Georgia (the Institutional Review Board of the National Center for Tuberculosis and Lung Diseases, Tbilisi, Georgia) and Switzerland (Ethikkommission Nordwest- und Zentralschweiz).

### Definitions

**A case of recurrent TB–**was defined as subsequent occurrence of the disease due to relapse or reinfection, after declaring the patient as clinically cured based on WHO guidelines [1]. **Cured–**bacteriologically confirmed TB patients were declared as “cured” in case of smear/culture negative result in at least once or in the last month of the treatment [13]. **Lost to follow-up**–patients who did not initiate or interrupted the treatment process for at least two following months [13]. **Relapse–**reactivation of endogenous infection that was not eliminated during treatment of a previous episode of TB [3]. **Reinfection**–re-occurrence of TB disease caused by *M*. *tuberculosis* strain distinct from the strain that caused the previous episode of TB disease [3].

### Case definition and epidemiological data

Patients were considered eligible for the study in case of recurrent MDR TB episode during the period 2014–2016, while being declared cured/successfully treated or lost to follow-up for the first/second TB episode.

Epidemiological data from both episodes was collected, when available. For statistical analysis, epidemiological variables which are biologically plausible potential risk factors for reinfection or relapse were considered.

### Bacteriology

The *M*. *tuberculosis* cultures from the primary and secondary episodes, stored at -80°C, were obtained from the Georgian NRL isolate collection. Frozen samples were thawed and sub-cultured on Lowenstein-Jensen medium. High quality DNA was extracted using the CTAB method as described previously [14]. DNA was stored at -20°C before being used for genotyping.

### MIRU-VNTR genotyping

Relapse and reinfection were differentiated using MIRU-VNTR genotyping; samples from both clinical episodes (pairs) were typed using the 24 loci panel as described [15]. Positive and negative controls were included in each PCR reaction, as H37Rv and H_2_O, respectively. Double allele results were confirmed with two independent PCRs. The 24 loci results were converted into numerical codes based on an allelic table as published by Supply *et al* [15]. The 24-digit profiles were compared using **www.miru-vntrplus.org**. Based on the results, each strain pair was defined as relapse or reinfection. Strain pairs with the same MIRU24 pattern or one locus of difference were considered as reflecting relapse. MIRU24 patterns differing by two or more loci between the two strains in a pair were defined as reinfection.

Double alleles in two or more loci were considered mixed infections and excluded from further analysis. Cases with double alleles in one locus were attributed to an event of intra-patient microevolution and considered a case of relapse [3].

### Statistical analysis

STATA v14.0 was used for statistical analysis. To compare categorical variables Fisher`s exact test was used and quantitative variables were compared using the Wilcoxon rank sum test. In all statistical comparisons, the significance level was set to 0.05. Logistic regression was used to assess associations between the odds of relapse / re-infection and the potential predictor variables gender, age, incarceration status during the primary or recurrent case, smear microscopy, HIV, number of people in household and smoking status were considered as independent variables. These predictor variables were considered one by one, in univariable models, and upon adjustment for age and imprisonment in multivariable models.

## Results

### Description of the study population

**Fig 1:**
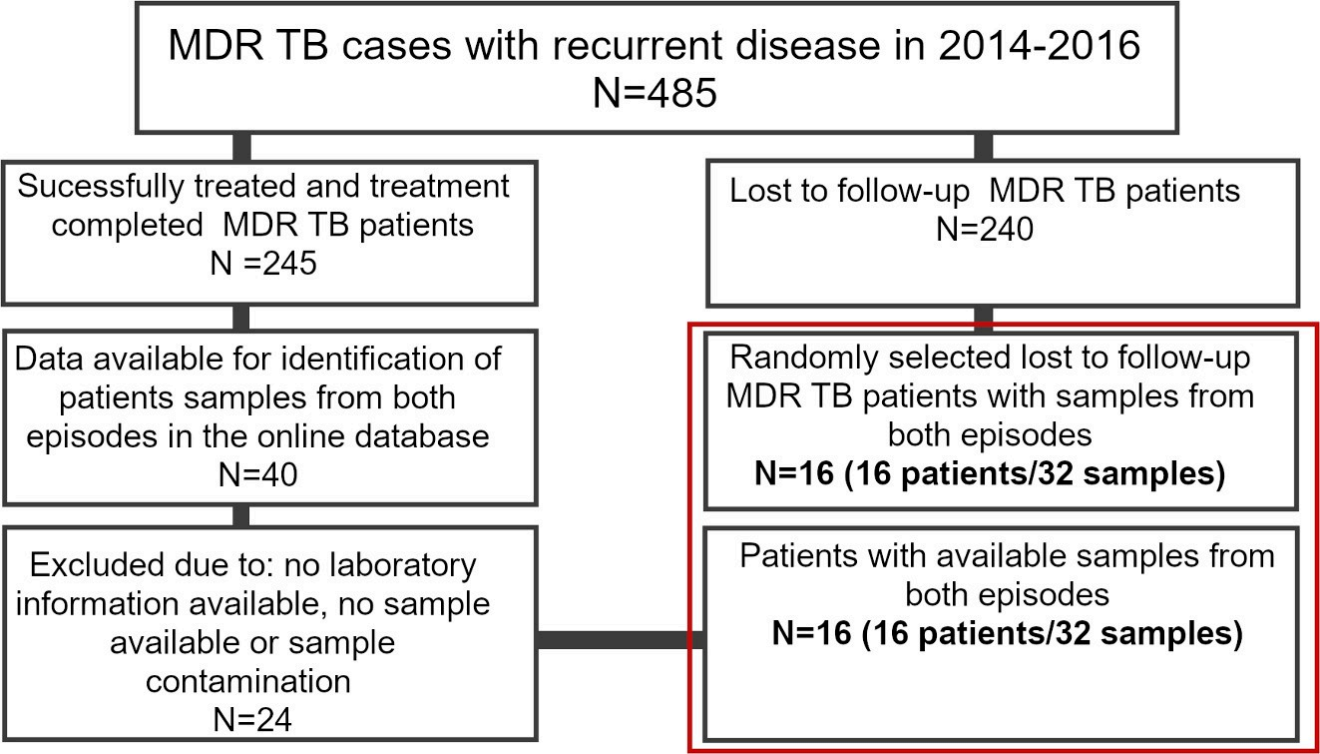
Flow diagram of selecting study participants.

From a total of 1,361 MDR TB cases enrolled in 2^nd^-line treatment between 2014 and 2016 in Georgia, recurrent TB was detected in 485 (35.6%) patients, of whom 245 (50.5%) were successfully treated (treatment completed and cured) in the past, and 240 cases (49.5%) were reported as lost to follow-up. Epidemiological records included information for 40 (16.3%) of the successfully treated patients (Fig 1). From the total of 40, after exclusion due to missing laboratory data, missing specimen, or sample contamination, we ended up with a complete data set of 16 MDR-TB patients who were successfully treated (and 32 paired *M*. *tuberculosis* isolates). An additional 16 patients from the “lost to follow up” group were randomly selected and included in the study for comparison. Hence, a total of 32 patients (64 paired samples) were included in the study (Fig 1).

### MIRU-VNTR typing results: Relapse vs. reinfection

Based on our MIRU24 typing results of the 64 paired *M*. *tuberculosis* isolates, 25/32 (78.1%) MDR TB patients had the same genotype in both paired isolates and were classified as relapse, while 5/32 (15.6%) showed different MIRU patterns and thus evidence of exogenous reinfection with a different strain. Two patients (6.3%) were classified as harboring a mixed infection.

### Comparing patient characteristics across patient groups

After excluding the two patients with mixed infections, further analysis was performed using clinical and demographical data for the remaining 30 patients (Table 1). The median age of these patients was 37.5 (Inter Quartile Range (IQR) = 28–43 years) and 41.5 (IQR = 29–47 years), for the first and recurrent TB episodes, respectively. In the relapse group, the median age at the first episode was 41 years (IQR = 33–45 years) years and for the recurrent case 44 years (IQR = 35–49 years). In the reinfection group, patients’ median age was 30 years (IQR = 26–42 years) at the first episode and 32 years (IQR = 28–43 years) at the second (i.e. reinfection) episode (Table 1). In the civil sector, relapse was observed in 20 (95.2%) patients out of 21. By contrast in the penitentiary system, 5 (55.6%) out of the 9 patients were due to relapse, while 4 (44.4%) were due to reinfection (Fisher’s Exact Test, P = 0.004) (Table 1).

**Table 1:** Characteristics of the relapse/reinfection cases defined by MIRU-VNTR typing.

Characteristics		Total No.	Relapse *n* (%)	Reinfection *n* (%)	P* value
Recurrence		30	25 (83.3)	5 (16.7)	
Sex	Female Male	5 25	5 (100) 20 (80)	0 5 (18.5)	0.37
Age at first episode	<30 31–40 41–50 >50	11 6 11 2	10 (90.9) 6 (100) 8 (72.7) 1 (50)	1 (9.1) 0 3 (27.3) 1 (50)	0.18
Age at second episode	<30 31–40 41–50 >50	9 5 12 4	9 (100) 4 (80) 9 (75) 3 (75)	0 1 (20) 3 (25) 1 (25)	0.28
Group	Cured Lost to follow-up	15 15	11 (73.3) 14 (93.3)	4 (26.7) 1 (6.7)	0.33
Origin	Civil Prison	21 9	20 (95.2) 5 (55.6)	1 (4.8) 4 (44.4)	0.004
Smear ^a^	Positive Discrepant Negative Unknown	14 13 2 1	12 (85.7) 10 (76.9) 2 (100) 1 (100)	2 (14.3) 3 (23.1) 0 0	0.75
HIV	Positive Negative Unknown	1 24 5	1 (100) 21 (87.5) 3 (60)	0 3 (12.5) 2 (40)	0.27
No. of people in household	0–2 3–5 ≥6 Unknown	6 6 12 6	5 (83.3) 10 (83.3) 5 (83.3) 2 (100)	1 (16.7) 2 (16.7) 1 (16.7) 0	0.57
Smoking	Yes No	16 10	13 (81.2) 9 (90)	3 (18.8) 1 (10)	0.65

*For categorical variables Fisher`s exact test was used. Quantitative variables were analyzed with Wilcoxon rank sum test.

^a^ Smear results from both episodes were compared, positive indicates Acid Fast Bacilli (AFB) (+) in both samples, discrepant refers to positive and negative results for either first or second sample, negative was defined with AFB(-) for both episodes.

Finally, we were interested in the median time to relapse in both groups of patients—successfully treated (including treatment completion) and lost to follow-up. The median time after successful treatment and treatment completion was 49 months (26.7 months; IQR 22.6–89.4 months), while for the lost-to-follow-up patients, the median period until relapse was, as expected because of the interruption in treatment, much shorter—17.9 months (20.5 months, IQR 5.9–56.2 months).

### Predictors for TB recurrence

Potential predictors for TB relapse and reinfection were analyzed using logistic regression (Table 2). Variables were adjusted for age and origin; odds ratios and CI were calculated. Odds ratios were obtained for lost to follow-up status, smear result, culture conversion, origin, smoking and number of people in the household, but no variable was statistically significant except imprisonment with high OR for reinfection (P-value = 0.03) (Table 2).

**Table 2:** Potential predictors of TB relapse and reinfection.

Factors associated with relapse							
Characteristics	N(%)30	Un-adjusted OR (95%CI*)	P-value	Adjusted OR (95%CI)^1^	P-value	Adjusted OR (95%CI)^2^	P-value
Lost to follow-up	15(50%)	5.1(52.3–0.5)	0.17	4.1 (44-0-37)	0.25	3.8 (47.9-0-29)	0.31
Smear positive	14(46.7%)	1.4 (9.8–0.2)	0.74	1.1 (8.74–0.15)	0.9	0.4 (5.7–0.03)	0.5
Culture conversion	18(60%)	1 (0.14–7.1)	1	1.21 (9.38–0.16)	0.86	2.86 (39.6–0.21)	0.43
Factors associated with reinfection							
Prison	18(60%)	16 (1.45–176.5)	0.02	51.93 (1.43–1891.5)	0.031	NA	NA
Smoker	16/26(61.5%)	2.08 (0.18–23.3)	0.55	3.3 (0.23–47.66)	0.38	1.64 (0.11–23.88)	0.72
Household members >2	22/28 (78.6%)	1.11 (0.1–12.31)	0.93	3.32 (0.16–70.11)	0.44	NA	NA

*CI = confidence interval

^1^—Adjusted OR (95%CI) for age

^2^—Adjusted OR (95%CI) for origin

Lost to follow-up patients, patients with positive smear result and culture conversion were associated with relapse, but neither was statistically significant. While having been imprisoned, smoking habit and more than two cohabitants were positively associated with re-infection, but only the association with having been imprisoned was statistically significant.

## Discussion

In this study, we used 24 loci based MIRU-VNTR typing to differentiate between relapse and reinfection in 32 patients with recurrent MDR-TB. Our results showed that the majority (>95%) of patients with recurrent MDR-TB from the civilian sector were due to relapse, whereas in patients with a history of incarceration, about half (44%) were due to reinfection.

The differentiation between relapse and reinfection in recurrent TB cases has major implications for the definition of national control measures. In the case of reinfection, prevention measures will need to be more inclusive at national level and require reducing TB transmission along with improving early detection of cases [2,16]. On the other hand, preventing relapse, which mostly affects individual patients, requires strengthening treatment adherence in patient populations at high risk of relapse and more patient-oriented care [17].

Until now, all recurrent TB cases in Georgia were classified as “relapse” by the national surveillance program. This classification, as our data suggests, fails to describe the true nature of the infection in a substantial proportion of patients. Based on our data, 16.7% of recurrent cases were due to reinfection, and these were significantly associated with a specific high risk population, i.e. prisoners. Timely diagnosis of TB disease is a crucial part of controlling TB transmission in penitentiary system, while delay in diagnosis leads to an increased risk of TB transmission [18]. Currently, the penitentiary system in Georgia provides an active TB screening program with questionnaires, followed by Xpert MTB/RIF test in case of disease suspicion. Regardless of the sharp decrease of TB cases in prison (National Surveillance Program, unpublished data) challenges still arise, in addition to the lack of convenient tools for controlling the disease [19,20]. Although our study was not directly focusing patients in prisons, our data shows that reinfection in TB is a significant problem in the penitentiary system that should be addressed.

Compared to drug susceptible TB, treatment for MDR-TB is long and more complex. Treatment for such patients includes second-line drugs that are known for their severe toxic side effects [21]. Increased risk of recurrence due to relapse in MDR-TB patients suggests that the current MDR-TB treatment might not be enough to completely eliminate the bacteria [22–24]]. The percentage of MDR-TB cases in previously treated TB patients in Georgia has increased from 31% in 2010 to 38% in 2016, followed by a decrease to 22% in 2018 (National Surveillance Program, unpublished data), indicating the presence of a reservoir for drug-resistant bacteria in the country, partially due to incomplete treatment. Surprisingly, we did not find a statistically significant association between relapse and “lost to follow-up”, but this could be due to the small sample set included in our study. We found no association between the number of people in households and risk of reinfection.

We managed to distinguish between relapse and reinfection in most of our cases. However, reinfection with the same strain of *M*. *tuberculosis* might suggest relapse, rendering the differentiation between relapse and reinfection more challenging. While MIRU-VNTR is still a widely used genotyping tool, whole genome sequencing (WGS) provides higher resolution to differentiate between closely related *Mycobacterium tuberculosis* strains. Due to the high costs and complex data analysis, using WGS is still limited, but gradually becoming more affordable, and thus likely to replace other genotyping methods in the near future [25].

One of the limitations of the study is the small sample size, leading to the large confidence intervals in our estimates. However, data from similar incidence settings show similar proportions of relapse and reinfection [9,26–28], and therefore we do not expect these proportions to change extensively, even if we included a larger sample size.

In conclusion, MIRU-VNTR typing base on 24 loci was implemented successfully in Georgia as a tool for differentiating between recurrent MDRTB caused by relapse versus reinfection. Our data revealed that relapse is a major contributor to problem of recurrent TB in Georgia. Despite the recent increases in resources made available to the National Tuberculosis Program (NTP) to decrease TB incidence countrywide, our data highlights the need for improved treatment completion and a reduction in the number of patients who were lost to follow-up.

## Supporting information

S1 Appendix MIRU-VNTR typing results. (XLSX)

## References

[1] WHO. Global tuberculosis report World Health Organization; 2018.

[2] Williams M, Müller B, Uys P, Victor TC, Warren RM, Gey van Pittius NC. Chapter 9: Tuberculosis recurrence: exogenous or endogenous? In: Antituberculosis Chemotherapy. Donald PR, van Helden PD(eds). Prog Respir Res. Basel, Karger, 2011, vol 40, pp 73–80

[3] McIvor A, Koornhof H, Kana BD. Relapse, re-infection and mixed infections in tuberculosis disease. Pathog Dis. 2017;75 10.1093/femspd/ftx020 28334088

[4] Lan NTN, Lien HTK, Tung LB, Borgdorff MW, Kremer K, van Soolingen D. Mycobacterium tuberculosis Beijing Genotype and Risk for Treatment Failure and Relapse, Vietnam. Emerg Infect Dis. 2003;9: 1633–1635. 10.3201/eid0912.030169 14720411 PMC3034339

[5] Mirsaeidi M, Sadikot RT. Patients at high risk of tuberculosis recurrence Int J Mycobacteriol. 2018 Jan-Mar;7(1):1–6. 10.4103/ijmy.ijmy_164_17 29516879

[6] Korhonen V, Smit PW, Haanperä M, Casali N, Ruutu P, Vasankari T, et al Whole genome analysis of Mycobacterium tuberculosis isolates from recurrent episodes of tuberculosis, Finland, 1995–2013. Clin Microbiol Infect Off Publ Eur Soc Clin Microbiol Infect Dis. 2016;22: 549–554. 10.1016/j.cmi.2016.03.014 27021423

[7] Parvaresh L, Crighton T, Martinez E, Bustamante A, Chen S, Sintchenko V. Recurrence of tuberculosis in a low-incidence setting: a retrospective cross-sectional study augmented by whole genome sequencing. BMC Infect Dis. 2018;18 10.1186/s12879-017-2906-7 29879906 PMC5992641

[8] Dale KD, Globan M, Tay EL, Trauer JM, Trevan PG, Denholm JT. Recurrence of tuberculosis in a low-incidence setting without directly observed treatment: Victoria, Australia, 2002–2014. Int J Tuberc Lung Dis Off J Int Union Tuberc Lung Dis. 2017;21: 550–555. 10.5588/ijtld.16.0651 28399970

[9] Uys PW, van Helden PD, Hargrove JW. Tuberculosis reinfection rate as a proportion of total infection rate correlates with the logarithm of the incidence rate: a mathematical model. J R Soc Interface. 2009;6: 11–15. 10.1098/rsif.2008.0184 18577502 PMC2610322

[10] Unis G, Ribeiro AW, Esteves LS, Spies FS, Picon PD, Dalla Costa ER, et al Tuberculosis recurrence in a high incidence setting for HIV and tuberculosis in Brazil. BMC Infect Dis. 2014;14: 548 10.1186/s12879-014-0548-6 25338623 PMC4215011

[11] Takarinda KC, Harries AD, Srinath S, Mutasa-Apollo T, Sandy C, Mugurungi O. Treatment outcomes of adult patients with recurrent tuberculosis in relation to HIV status in Zimbabwe: a retrospective record review. BMC Public Health. 2012;12: 124 10.1186/1471-2458-12-124 22329930 PMC3305664

[12] Interrante JD, Haddad MB, Kim L, Gandhi NR. Exogenous Reinfection as a Cause of Late Recurrent Tuberculosis in the United States. Ann Am Thorac Soc. 2015;12: 1619–1626. 10.1513/AnnalsATS.201507-429OC 26325356 PMC4724895

[13] World Health Organization. Definitions and reporting framework for tuberculosis. 2013 Dec p. 40. Report No.: WHO/HTM/TB/2013.2.

[14] De Almeida IN, Da Silva Carvalho W, Rossetti ML, Costa ERD, De Miranda SS. Evaluation of six different DNA extraction methods for detection of Mycobacterium tuberculosis by means of PCR-IS6110: preliminary study. BMC Res Notes. 2013;6: 561 10.1186/1756-0500-6-561 24373461 PMC3891981

[15] Supply P, Allix C, Lesjean S, Cardoso-Oelemann M, Rüsch-Gerdes S, Willery E, et al Proposal for Standardization of Optimized Mycobacterial Interspersed Repetitive Unit-Variable-Number Tandem Repeat Typing of Mycobacterium tuberculosis. J Clin Microbiol. 2006;44: 4498–4510. 10.1128/JCM.01392-06 17005759 PMC1698431

[16] Afshar B, Carless J, Roche A, Balasegaram S, Anderson C. Surveillance of tuberculosis (TB) cases attributable to relapse or reinfection in London, 2002–2015. PLOS ONE. 2019;14: e0211972 10.1371/journal.pone.0211972 30768624 PMC6377187

[17] Cazabon D, Alsdurf H, Satyanarayana S, Nathavitharana R, Subbaraman R, Daftary A, et al Quality of tuberculosis care in high burden countries: the urgent need to address gaps in the care cascade. Int J Infect Dis IJID Off Publ Int Soc Infect Dis. 2017;56: 111–116. 10.1016/j.ijid.2016.10.016 27794468 PMC5346036

[18] Droznin M, Johnson A, Johnson AM. Multidrug resistant tuberculosis in prisons located in former Soviet countries: A systematic review. PLOS ONE. 2017;12: e0174373 10.1371/journal.pone.0174373 28334036 PMC5363920

[19] Shamputa IC, Jugheli L, Sadradze N, Willery E, Portaels F, Supply P, et al Mixed infection and clonal representativeness of a single sputum sample in tuberculosis patients from a penitentiary hospital in Georgia. Respir Res. 2006;7: 99 10.1186/1465-9921-7-99 16846493 PMC1538999

[20] Gegia M, Kalandadze I, Madzgharashvili M, Furin J. Developing a human rights-based program for Tuberculosis control in Georgian prisons.Health Hum Rights. 2011 12 15;13(2):E73–81. 22773034 PMC3734935

[21] WHO. WHO treatment guidelines for multidrug and rifampicin-resistant tuberculosis. 2018.

[22] Bestrashniy JRBM, Nguyen VN, Nguyen TL, Pham TL, Nguyen TA, Pham DC, et al Recurrence of tuberculosis among patients following treatment completion in eight provinces of Vietnam: A nested case-control study. Int J Infect Dis. 2018;74: 31–37. 10.1016/j.ijid.2018.06.013 29944930

[23] Millet J-P, Shaw E, Orcau A, Casals M, Miró JM, Caylà JA, et al Tuberculosis recurrence after completion treatment in a European city: reinfection or relapse? PloS One. 2013;8: e64898 10.1371/journal.pone.0064898 23776440 PMC3679149

[24] Verver S, Warren RM, Beyers N, Richardson M, van der Spuy GD, Borgdorff MW, et al Rate of Reinfection Tuberculosis after Successful Treatment Is Higher than Rate of New Tuberculosis. Am J Respir Crit Care Med. 2005;171: 1430–1435. 10.1164/rccm.200409-1200OC 15831840

[25] Meehan CJ, Goig GA, Kohl TA, Verboven L, Dippenaar A, Ezewudo M, et al Whole genome sequencing of Mycobacterium tuberculosis: current standards and open issues. Nat Rev Microbiol. 2019;17: 533–545. 10.1038/s41579-019-0214-5 31209399

[26] Wang J-Y, Lee L-N, Lai H-C, Hsu H-L, Liaw Y-S, Hsueh P-R, et al Prediction of the tuberculosis reinfection proportion from the local incidence. J Infect Dis. 2007;196: 281–288. 10.1086/518898 17570116

[27] Caminero JA, Pena MJ, Campos-Herrero MI, Rodríguez JC, Afonso O, Martin C, et al Exogenous Reinfection with Tuberculosis on a European Island with a Moderate Incidence of Disease. Am J Respir Crit Care Med. 2001;163: 717–720. 10.1164/ajrccm.163.3.2003070 11254530

[28] Gadoev J, Asadov D, Harries AD, Parpieva N, Tayler-Smith K, Isaakidis P, et al Recurrent tuberculosis and associated factors: A five—year countrywide study in Uzbekistan. PLOS ONE. 2017;12: e0176473 10.1371/journal.pone.0176473 28472053 PMC5417503

